# Using Human-Centered Design to Develop a Program to Engage South African Men Living With HIV in Care and Treatment

**DOI:** 10.9745/GHSP-D-21-00239

**Published:** 2021-11-29

**Authors:** Cal Bruns

**Affiliations:** a Matchboxology, Cape Town, South Africa.

## Abstract

**Background:**

Although HIV care is widely available in South Africa, men are less likely than women to know their HIV status, begin treatment upon diagnosis, and adhere to treatment. Men are also more likely to die from causes related to HIV compared to women. To overcome this inequity, tailored approaches designed with men’s specific needs are required.

**Methods:**

As part of the Testing and Treatment for Men project, a 3-year multicomponent, mixed-methods study that aimed to better understand young South African men’s needs concerning HIV testing and treatment, we used human-centered design (HCD) methods to identify, develop, and pilot test a male-friendly peer-support intervention. We engaged key stakeholders in the development of several prototype interventions to improve men’s engagement across the HIV cascade, one of which was selected for further development.

**Results:**

Use of HCD methods, informed by the results of our team’s prior qualitative and quantitative research, led to the development of Coach Mpilo, a peer-support intervention to engage and retain men living with HIV in South Africa in care and treatment. By designing with empathy for the lived experiences of these men, our intervention achieved the authenticity and credibility required to build trusting relationships in the affected communities. Preliminary results of Coach Mpilo have demonstrated a high level of acceptability by men living with HIV as well as key government and other implementing partners.

**Conclusion:**

HCD as a critical ingredient became an integral and essential component in developing and field-testing the Coach Mpilo pilot. The key elements of our HCD approach that resulted in the successful generation of the Coach Mpilo pilot intervention and its implementation were understanding and addressing the specific issues identified by men living with HIV and leveraging newly created empathy with men as an approach to solving the issues collaboratively and innovatively.

## Abstract (precis)

Human-centered design (HCD) is a useful methodology for understanding the lived realities, needs, and preferences of men living with HIV and engaging them in the design and pilot of a peer-support program to support their engagement in care.

## Teaser

**Key Messages:**

- HCD’s structured, collaborative problem-solving process inspired the partners and allowed integration with multiple research methodologies. HCD excited seasoned HIV prevention and treatment veterans in the project’s advisory group. The segmentation of at-risk male archetypes allowed men’s attitudes, behaviors, and challenges to be seen through a human lens and informed HCD co-creation problem statements.
- HCD, paired with qualitative elicitation methods, allowed a deeper empathy with at-risk men and health professionals, ensuring the design of prototypes that addressed specific needs of men were desirable for them, as well as being feasible and viable for implementers.
- By integrating South African organizations, the Department of Health, and other funders into a project conceived with design thinking as an ingredient, our co-created innovations were primed for program integration and rapid scale-up with ongoing improvement through iteration.

**Key Implications:**

Program managers and policy makers should consider using HCD approaches when designing or preparing to scale up interventions. In addition to informing program design, the implementation of HCD will foster stakeholder empathy, trust, and program “ownership” that will optimize acceptability, effectiveness, and sustainability of the intervention.

## BACKGROUND

Despite improvements in the availability of HIV testing, care, and treatment programs across sub-Saharan Africa, men’s engagement across the HIV cascade is suboptimal.^1^^–^^7^ In South Africa, of the estimated 7.5 million adults (15 years and older) living with HIV in 2020, approximately 36% were adult men.^8^ Compared to their female counterparts, South African men are less likely to know their HIV status, begin treatment upon diagnosis, or adhere to treatment.^6^^–^^8^ Consequently, South African men living with HIV are more likely to die of AIDS-related causes.^9^^,^^10^ To reach the global UNAIDS fast-track targets of 95-95-95 by the end of 2030—whereby 95% of people living with HIV know their status, 95% of those who know they are positive are on antiretroviral therapy (ART), and 95% of those on ART are virally suppressed—^11^ it is imperative that men are better engaged across the HIV care cascade. Effective strategies are needed to identify, engage, and retain men living with HIV in care to improve their health and to prevent ongoing transmission to their partners.

With funding from the Bill & Melinda Gates Foundation, our consortium, including members with expertise in population health research and evaluation (Population Services International and Ipsos) and program design (Matchboxology), implemented Testing and Treatment for Men (TTM), a 3-year project aiming to increase uptake of HIV testing services by young South African men at high risk of acquiring HIV as well as initiation of ART for those newly diagnosed and those who had dropped out of care. A mixed-methods approach was used to understand men’s decisions and behaviors related to HIV testing, prevention, and treatment and to identify different segments of young men to enable better-tailored interventions. A human-centered design (HCD) approach was used to explore strategies for reaching each segment with HIV prevention, testing, and treatment services more effectively.

A human-centered design approach was used to explore strategies for reaching each segment with HIV prevention, testing, and treatment services more effectively.

HCD is a dynamic approach that engages end-users and key stakeholders to develop tailored, usable products, programs, or systems.^12^^–^^14^ For the past decade, there has been increasing adoption and integration of HCD practices in development initiatives, particularly in global health programming.^15^^,^^16^ Recently, the Design for Health community—launched by the Bill & Melinda Gates Foundation and the Center for Innovation and Impact in the U.S. Agency for International Development’s (USAID) Bureau for Global Health—have defined the following 3 models by which design methods and mindsets can be applied in the development of global health solutions.

1. Spark: As a **spark,** design is used as an inspiration method to reframe the understanding of existing problems to make way for innovations. This might occur during structured brainstorming sessions focused on a clearly defined challenge to encourage teams to bring new ideas to the table.
2. As an **ingredient,** design can be used to improve an existing product or service by conducting rapid research to better understand the improvements needed and conducting a series of “trial and error” testing sessions to determine which ideas should be incorporated to improve the user experience of the existing product or service.
3. Using design in an **end-to-end** approach requires full adoption of design thinking and methods throughout solution development processes including conducting design research to reveal new user insights relevant to the challenge or need, codesigning solutions in partnership with key stakeholders, gathering feedback through prototyping and testing of ideas, and continued testing and refinement of the idea throughout implementation.

In this Supplemental issue of *Global Health: Science and Practice*, each case study will demonstrate the ways design was used either as a spark, ingredient, or an end-to-end approach to drive innovative ways of addressing complex global health challenges. In this article, we describe the process of using a design thinking approach as a critical ingredient for developing prototype interventions, followed by the selection of and pilot testing Coach Mpilo, a peer-support intervention designed to increase uptake of HIV testing, prevention, and linkage to care and treatment among young men in South Africa.

## METHODS

**FIGURE 1:**
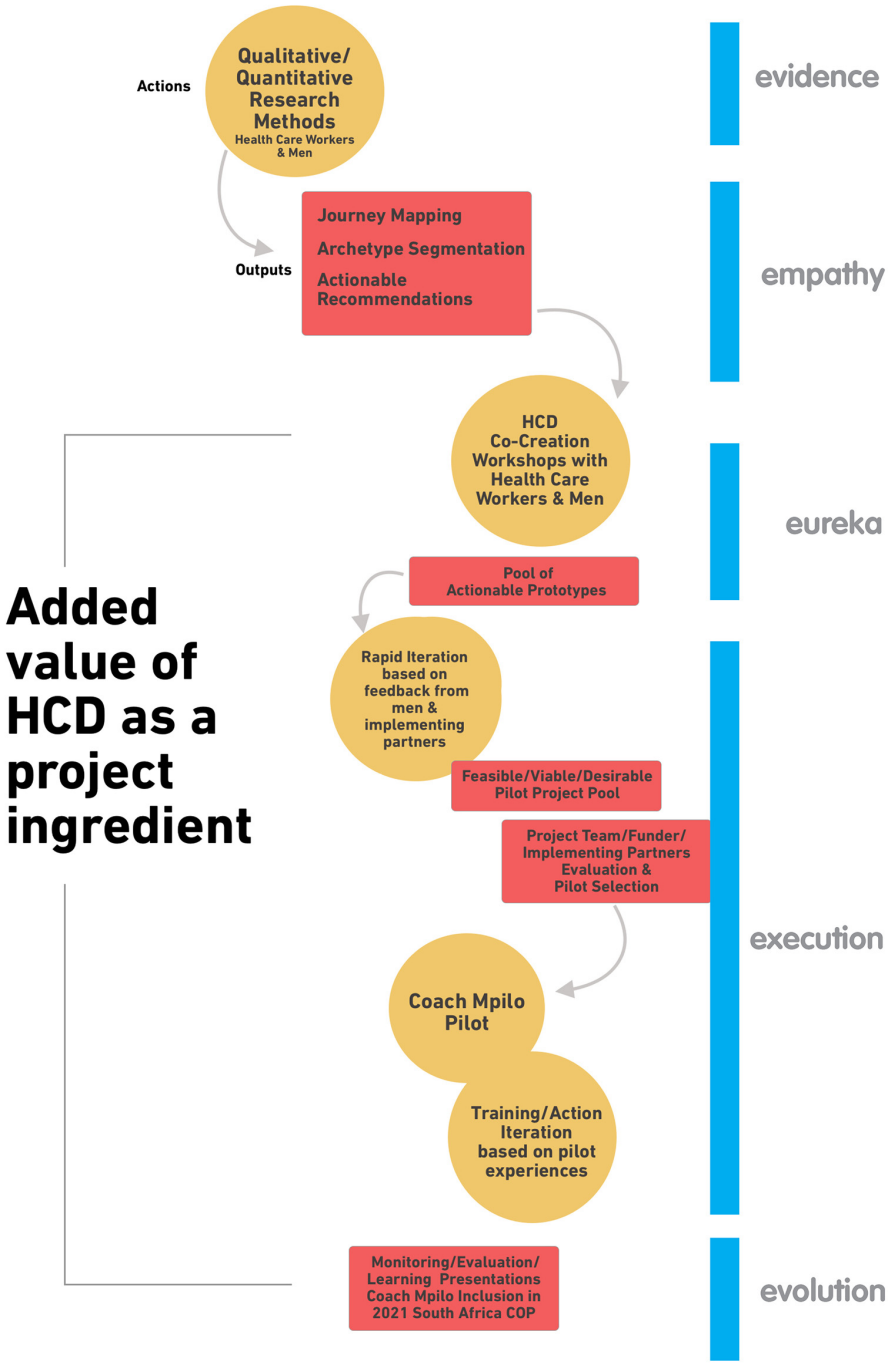
Human-Centered Design Process Used to Develop Prototypes of Interventions to Increase HIV Testing and Update of Antiretroviral Therapy Among Men

The TTM project was conducted in 3 districts in South Africa with high HIV prevalence—Ehlanzeni and Gert Sibande (Mpumalanga Province) and Ugu (KwaZulu-Natal Province). We used an HCD approach to develop prototypes of interventions to achieve the aims of the TTM (i.e., increase HIV testing and uptake of ART among men who were newly diagnosed with HIV or who had previously dropped out of care). The design process was informed by input from an advisory group, as well as results from qualitative and quantitative studies led by members of our consortium. The design process was iterative (Figure 1). We briefly describe the components of the project.

### Advisory Group Consultation

Advisory group (AG) members comprised national, provincial, and district Department of Health officials; national and local implementing partners; HIV-focused funders; representatives of normative bodies including the World Health Organization and UNAIDS; and civil society organizations. We began with a “learning and listening workshop” augmented with one-on-one meetings with selected members. These were highly interactive opportunities to: (1) share learnings from previous successes and failures, (2) understand what AG members defined to be relevant academic or practical evidence based on lived experiences working with men, and (3) explore each participating organization’s barriers, attitudes, motivations, and deliverables. Members were formally engaged at key design decision milestones across the project timeline.

### Qualitative Data Collection

We recruited and enrolled health care providers and HIV-positive and HIV-negative men aged 25–34 years in high-HIV prevalence districts in Mpumalanga and KwaZulu-Natal Provinces to participate in qualitative data collection activities, including full-day ethnographic shadowing with men (n=18) and health care providers (n=4), as well as in-depth interviews with men (n=58) and health care providers (n=64). Ethnographic shadowing involved following and observing participants during their usual routines and asking them to interpret actions through informal interviews. All ethnographic work was conducted by male interviewers in the respondents' home languages.

### Quantitative Data Collection

Details of the methods used have been previously presented.^17^ In brief, between October 2018 and January 2019, a cross-sectional study was conducted in 8 district municipalities in Mpumalanga and KwaZulu-Natal Provinces including from 2,019 Black African men aged 20–34 years with less than a university-level education. Participants completed a survey of demographic characteristics, sexual behavior, engagement with HIV testing and treatment services, alcohol use, HIV knowledge, mental health, and attitudes to gender equity. The study was informed by the Theoretical Domain Framework^18^ and analyzed following a protocol described by Sgaier et al.^19^

### Prototype Design

**FIGURE 2:**
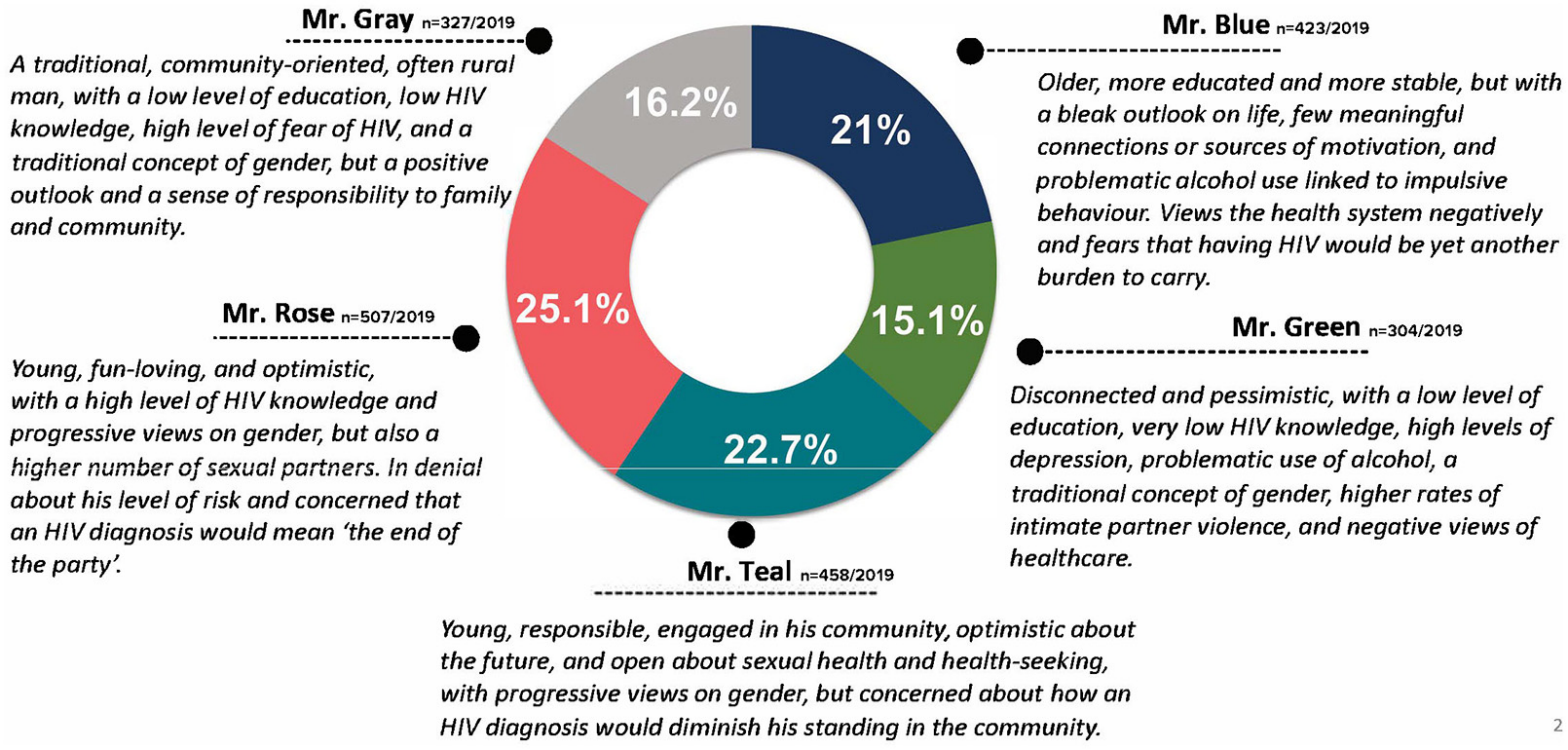
Characteristics of Male Archetypes Used in Segments That Incorporate Infection Attitudes, Beliefs, and Behaviors Related to HIV Testing and Treatment

The results of the qualitative and quantitative studies were presented to the core group of partners and funders in February 2019, when data were synthesized to produce descriptions of each segment that incorporated the salient characteristics and attitudes, beliefs, and behaviors related to HIV testing and treatment (Figure 2). Based on insights from the segmentation, actionable prototype interventions were generated by core team members through the use of various HCD methods,^13^ including brainstorming, “how might we” questions, role-play, visualization, theme identification, and exercises (Table 1).

**TABLE 1.** HCD Activities to Support the Co-design of Program Prototypes

**Activity**	**Participants**	**Description**	**Contribution to Program Design**
**Engage with AG**	National, provincial, and district DOH officials National and local implementing partners HIV-focused funders Local experts	Learning and listening workshop augmented with one-on-one meetings with selected members	– Learnings shared from previous successes and failures – Understand what AG members defined to be relevant academic or practical evidence based on lived experiences working with men – Explore each participating organization’s barriers, attitudes, motivations, and deliverables– The AG served as an expert resource with strategic checkpoints to change direction in real-time
**Video ethnography**	n=18 men; n=4 HCWs	1-day video “follow-alongs” in communities	Increased empathy for men’s self-reported interactions with community members and the health care system
**IDIs**	n=58 men; n=64 HCWs	2-hour IDIs in high HIV risk, hard-to-reach communities Journey mapping, a technique visualizing the health-seeking journey of at-risk men to deepen empathy and inform problem solving	– Provided men space and time to express feelings and insecurities that informed qualitative survey design– Highlighted logistical and emotional challenges of at-risk men in South Africa, drawing attention to friction points and barriers for confirmation via QS
**Quantitative surveys**	N=2,000 men aged 25–34 years; high school education or less	1-hour tablet-based surveys with random sample of men across 5 districts in KwaZulu-Natal and 3 districts in Mpumalanga	Generated statistically significant data points for robust segmentation analysis
**Segmentation of men**		Cluster analysis via modeling, options evaluation, and profiling based on solutions	Generated 5 distinct segments of men who gave new insights to seasoned implementers, informed problem statements and recruitment into co-creation process
**How might we? challenges**	n=32 workshop participants x 3 workshops	6 “How might we?” questions: A positive, actionable question that frames the challenge—a prompt used in co-creation workshops to focus participants on a specific topic and generate ideas	6 “How Might We?” focused brainstorming to solve very specific, granular issues facing 2 prioritized segments deemed most likely to produce the greatest impact across all 5 segments of men Generated ideas for strategies that would be responsive to the needs of 2 prioritized segments deemed most likely to produce the greatest impact across all 5 segments of men
**Role playing**	3 professional actors	5 monologues informed by segmentation and performed by professional actors from high-risk communities for overseas-based team members and advisory group	Significantly enhanced understanding of nuanced differences between 5 segments and helped inform the decision to focus prototyping on 2 specific segments of men
**Experience design**	32 workshop participants x 3 workshops	Focusing on the range of different health care access and service delivery touch points to reveal key gaps in quality service delivery that might otherwise fall through the cracks	Bringing together men, HCW from clinics, and district DOH staff as equal partners to design, rethink assumptions, perceive impossibilities, and unlock new ideas
**Co-creation prototyping**	n=32 workshop participants × 3 workshops; participants consisted of men from 2 segments (recruited using an Ipsos-generated typing tool), HCWs, and DOH staff Field testing storyboarded prototypes	Co-designing a variety and range of tangible engagement actions with users and implementers via storyboarding, which are believed to solve a specific “How might we?” challenge	Produced 20 diverse prototype concepts which were refined to 15 simple-to-understand men’s engagement and service design proposals shared with men in communities and implementation partners for further evaluation
**Rapid prototype feedback and iteration**		15 visualizations of new service designs and engagement initiatives were shared with a random sample of men across the project recruitment communities	Informed iterative design alterations to several of the prototypes, including changing the name of the coaches to *Coach Mpilo*
**Prototype evaluation/** **prioritization**		Prioritization ranking analysis based on 4 criteria: feasible/viable/desirable/speed to results	4 prototypes emerged as pilot finalists; *Coach Mpilo* selected as the first fielded pilot

Abbreviations: AG, advisory group; DOH, Department of Health; HCD, human-centered design; IDI, in-depth interviews.

**FIGURE 3:**
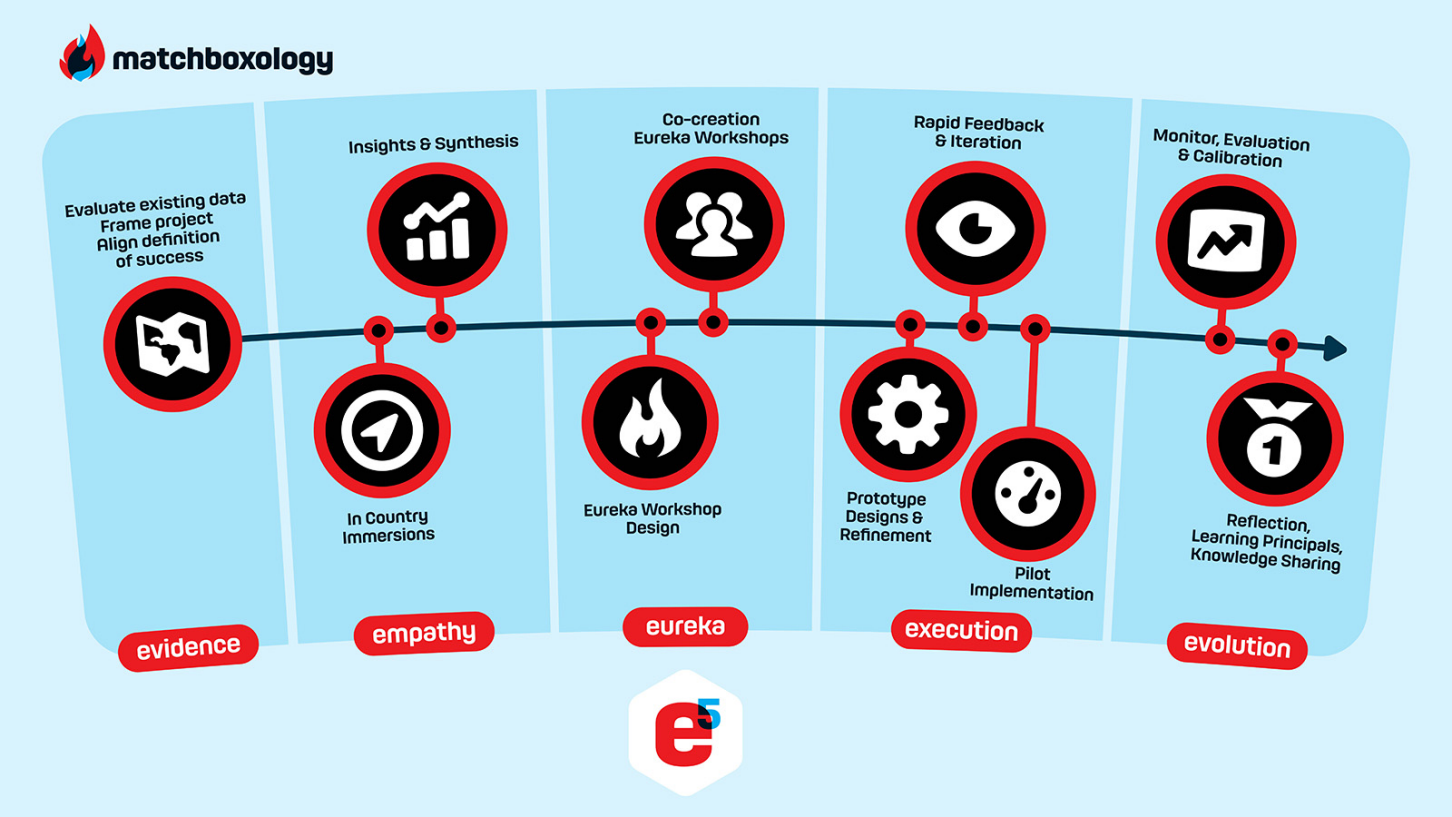
Matchboxology's 5-Step Human-Centered Design Process

Three 3-day residential workshops were held (1 in Nelspruit, Mpumalanga, and 2 in Durban, KwaZulu-Natal), each including up to 32 participants representing a diverse mix of stakeholders including male community participants, nurses, community leaders, and representatives from the provincial departments of health and implementing partners. Community participants were recruited by study staff members using a segmentation screening tool, developed by Ipsos, for “Mr. Green” and “Mr. Rose” types (Figure 2); potential participants were screened in short one-on-one interviews to ensure candidates had the capacity to express opinions and ideas within a workshop setting. Local clinic nurses and community leaders, district and provincial department of health officials, and AG members were also invited to participate in these workshops. Activities were organized around Matchboxology’s e5 HCD process (Figure 3), designing and calibrating solutions that are responsive to the needs of the end-user and focused on addressing the various challenges that emerged in qualitative interviews with men representing the archetypes represented by “Mr. Green” and “Mr. Rose” (Figure 2).

## RESULTS

### Qualitative and Quantitative Studies

Qualitative research revealed several key barriers and challenges, most significantly that: men are afraid, not stubborn or indifferent; they have no one they trust or feel safe to talk to; they associate HIV testing and treatment initiation with loss, not gain; and they anticipate a negative experience in the clinic and by others following disclosure of their HIV status. The quantitative segmentation study produced 5 subgroups or segments of men, based on their attitudes, beliefs, behaviors, and needs related to HIV testing and treatment (described in depth in Bell et al).^17^

Qualitative research revealed several key barriers and challenges such as men feel they have no one they trust or feel safe to talk to and they associate HIV testing and treatment initiation with loss, not gain.

## Prototype Design Activities

The AG recommended further development of prototype interventions focused on 2 of the 5 segments that were deemed the largest and most challenging: “Mr. Green” and “Mr. Rose” (Figure 2).

Workshop participants generated more than 20 solution concepts, converging on 4 broad themes that revealed the following needs:

- “Flip the script” on ART: HIV treatment can trigger negative emotional responses. We need to change those so that men can feel good about being on treatment. Example: Move from a daily reminder that “I’ve lost control” to a daily reminder that “I’m in charge.”
- Make HIV a collective challenge: Many men feel that having HIV is treated as a personal failure rather than a public health problem and that they are blamed and shamed rather than being supported. In reality, the strongest predictor of a man’s HIV risk is not his behavior but where he lives. HIV isn’t a personal failure, it’s a community challenge.
- Help men feel they are not alone. Treatment leaves many men feeling alone, afraid, and ashamed. Many men feel there can be no good life after an HIV diagnosis. But men also said they could accept advice and support from a man who is living with HIV and doing well.
- Make the clinic a more familiar and welcoming space.

**FIGURE 4:**
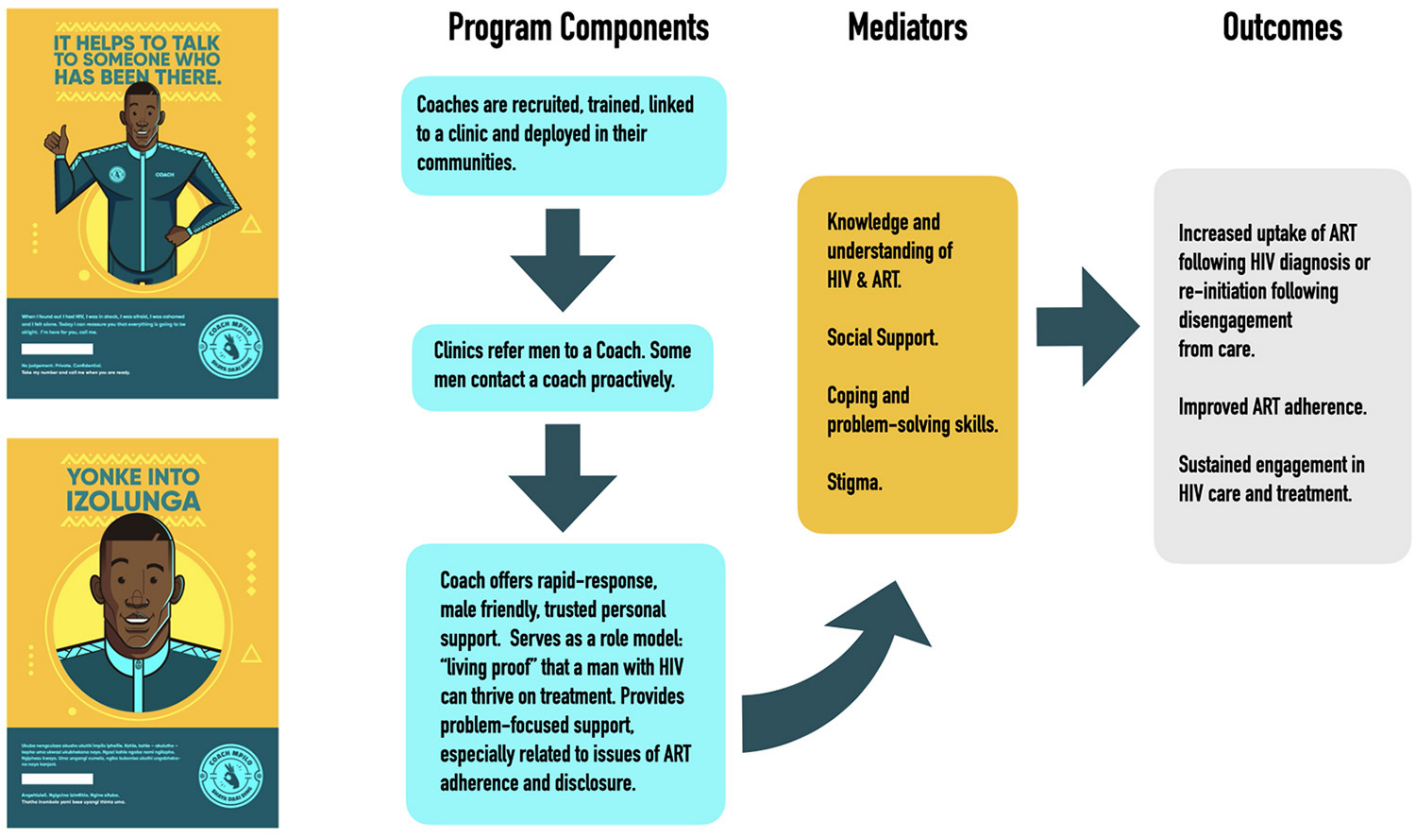
Coach Mpilo Program Conceptual Framework Abbreviation: ART, antiretroviral therapy.

In selecting among possible solutions to pilot, we selected Coach Mpilo (Mpilo means health in Zulu and is a play on the English word pill), as it responded to all of these identified needs. The prototype was fully developed in collaboration with men and other stakeholders. Details of the program may be found elsewhere.^20^ In brief, Coach Mpilo is a peer-support model that recruits and employs men living with HIV who are stable on ART as coaches of newly diagnosed men and men lost to follow-up (Figure 4). It was designed to resonate with men in several ways including breaking the isolation and paralysis that many men feel at diagnosis, giving men someone they can relate to and feel safe talking to, giving men “living proof” that a man can have HIV and live a normal life, supporting men to reimagine a positive future, and helping the coaches to reframe and reclaim their own identities. In addition to supporting men living with HIV to stay engaged in care and treatment, Coach Mpilo was also designed to leverage other key benefits (Table 2). Figure 4 provides an overview of the Coach Mpilo intervention and how it is designed to work.

**TABLE 2.** Additional Key Benefits Leveraged by *Coach Mpilo*

**Rapid response**	Coaches can be recruited in any community, trained in 4 days, and immediately deployed.
**Low cost**	Coaches are paid a modest salary and a transport/data stipend, and otherwise require minimal infrastructure or support.
**Relieving pressure on clinic staff**	Clinic staff can refer challenging cases to a coach.
**Stigma reduction**	Stigma is reduced among family and community members by providing living proof that a man with HIV can thrive on treatment.
**Leveraging the power of peer outreach**	Peer-outreach programs have been used in key populations programs for many years.
**Putting men living with HIV at the forefront of the response**	“Nothing about us without us.”

Since March 2020, the Coach Mpilo program has been implemented in Ehlanzeni and Gert Sibande Districts, Mpumalanga Province, and Ugu District, KwaZulu-Natal Province and has employed 120 coaches and reached over 3,700 men living with HIV. Preliminary data indicate that more than 90% of men are linked or returned to care within the first month of support and remain on treatment thereafter. HCD-driven piloting and rapid field iteration allowed course corrections to tailor the final prototype. The program has been met with a high level of acceptability by men, as well as key government, implementing, and funding partners. Coach Mpilo has been integrated by 4 implementing partners—Anova Health Institute, Aurum Institute, Centre for Communication Impact, and Wits Reproductive Health and HIV Institute—into current programming. Coach Mpilo has now been included in USAID’s 2021 country operating plan.

Preliminary data on the Coach Mpilo program indicate that more than 90% of men are linked or returned to care within the first month of support and remain on treatment thereafter.

## DISCUSSION

By integrating HCD principles as a key ingredient during the development, implementation, and evaluation of the Coach Mpilo pilot program, we found that an HCD approach ensured empathy in understanding how men living with HIV in South Africa experience the world and keeping them at the center of problem statements and engagement design processes. This depth of empathy also empowered decision makers in making informed decisions that would achieve better outcomes and allowed for robust, rapid evaluations of prototypes that emerged from the HCD methodologies because all parties had considered issues of desirability, feasibility, and viability from the beginning of the endeavor. The AG served as an expert resource with strategic checkpoints to change direction in real-time. The team incorporated inputs provided by the AG and other stakeholders while being mindful of potential implementation barriers and tailoring project components accordingly. Ethnographic and qualitative studies were a critical part of the HCD process as the results of those studies directly informed the co-creation sessions and prototype iterations.

By applying the HCD approach, we developed an intervention to retain men living with HIV in South Africa in care and treatment services. It was clear from this process that:

- HCD methods have the potential to create prototypes for programs that are highly desirable, feasible, and viable
- Applying HCD philosophies throughout stakeholder engagement helps to ensure both greater empathy among all parties and the “ownership” of health programs that will contribute strongly to their success and sustainability

The HCD process enabled our team to identify that the fundamental and unique value of the coach is that of trust. This trust was consistently protected within our design by the requirement that coaches come from **outside** of a clinic system that men have grown to distrust. Each coach had credibility because he is someone who has “been there with the virus”; this was articulated through the HCD process as the most valuable proposition in the prototype. Being a coach has also changed the self-esteem and community respect for the men who adopt this role. In addition, Coach Mpilo addresses a critical national issue of unemployment, giving work to men in a country with an unemployment rate of more than 30%.

The HCD process enabled our team to identify that the fundamental and unique value of the coach is that of trust.

Quality control throughout each aspect of the design process, data collection, and program implementation was critical to success. Maintaining prototype design fidelity required that coach positions be filled only by men living with HIV. By designing with empathy for the lived experiences of these men, our intervention achieved the authenticity and credibility required to build trusting relationships in the affected communities. HCD workshops stressed that coaches must be mentors who have “lived the journey”—a practice that resulted in improved engagement in retention in HIV care and treatment services. The performance of the coaches in the pilot revealed the impact of reducing stigma, which potentially accelerated ART uptake and adherence.

The key to the success of the design process and program pilot of Coach Mpilo was the influence of an “HCD as ingredient” approach on the project philosophy and its application in the development of the intervention. The success of future iterations of Coach Mpilo will require applying HCD principles to the implementation and scale-up of the intervention. This will help to ensure that stakeholder empathy, trust, and program “ownership” are integrated into all aspects of program design and rollout, driving toward greater acceptability, success, and sustainability of the intervention.

“Mission creep” presents one of the most significant risks for scaling up Coach Mpilo. Funders may look for ways to leverage investments in Coach Mpilo to gather additional health burden data or perform other functions in their communities (e.g., TB or gender-based violence education). The coach’s value to the community and to the individual is as a trusted advisor independent from the traditional health care system, and adding roles beyond this core competency risks compromising program impact and data quality. Plans for scale-up of Coach Mpilo will thus need to consider several key issues and potential challenges, including program ownership and accountability, more rigorous evaluation approaches, and long-term sustainability and commitments for funding.

## CONCLUSION

Deployed as an ingredient in this and similar projects, HCD can help to avoid errors based on top-down assumptions and biases and incorporate the lived experiences and perspectives of men living with HIV, as well as health care ecosystem expertise, into producing desirable and workable solutions that reduce the risk of failure when piloted.

Ultimately, our process generated deeply empathetic and highly pragmatic solutions by and for men living with HIV that clearly addressed their issues related to linkage to and retention in HIV care and treatment services. Every man’s life changed is a story, not just a statistic. By engaging key stakeholders in the design of our interventions, HCD helped to produce outcomes that matter.

We hope that the Coach Mpilo pilot can inform the integration of HCD that addresses health programs and interventions across diverse populations and health issues.

## Notes

**Peer Reviewed**
